# Heterogeneous Wireless Networks for Smart Grid Distribution Systems: Advantages and Limitations

**DOI:** 10.3390/s18051517

**Published:** 2018-05-11

**Authors:** Tarek Khalifa, Atef Abdrabou, Khaled Shaban, A. M. Gaouda

**Affiliations:** 1 Computer Engineering Department, American University of the Middle East, PO 220 Dasman 15453, Kuwait; Tarek.khalifa@aum.edu.kw; 2 Department of Electrical Engineering, UAE University, Al-Ain, Abu Dhabi PO 15551, UAE; 3 Department of Computer Science and Engineering, Qatar University, Doha PO 2713, Qatar; khaled.shaban@qu.edu.qa; 4 Electrical Engineering Department, American University of the Middle East, PO 220 Dasman 15453, Kuwait; Ahmed.Gaouda@aum.edu.kw

**Keywords:** smart grids, distribution system, communication infrastructure, heterogeneous wireless networks

## Abstract

Supporting a conventional power grid with advanced communication capabilities is a cornerstone to transferring it to a smart grid. A reliable communication infrastructure with a high throughput can lay the foundation towards the ultimate objective of a fully automated power grid with self-healing capabilities. In order to realize this objective, the communication infrastructure of a power distribution network needs to be extended to cover all substations including medium/low voltage ones. This shall enable information exchange among substations for a variety of system automation purposes with a low latency that suits time critical applications. This paper proposes the integration of two heterogeneous wireless technologies (such as WiFi and cellular 3G/4G) to provide reliable and fast communication among primary and secondary distribution substations. This integration allows the transmission of different data packets (not packet replicas) over two radio interfaces, making these interfaces act like a one data pipe. Thus, the paper investigates the applicability and effectiveness of employing heterogeneous wireless networks (HWNs) in achieving the desired reliability and timeliness requirements of future smart grids. We study the performance of HWNs in a realistic scenario under different data transfer loads and packet loss ratios. Our findings reveal that HWNs can be a viable data transfer option for smart grids.

## 1. Introduction

Establishing real-time communication among a variety of power grid components at different voltage levels has been the focus of the communication research community for a while. Power utility companies have had a successful experience with the supervisory control and data acquisition (SCADA) systems in monitoring and securing the high voltage (HV) transmission part of the grid. Communication technologies have also supported similar functions at the more component-dense distribution part of conventional power grids. However, particularly for the distribution part, smart grids expect much higher support from communication technologies. This is required for upgrading the system smart functions such as self-healing, where a system controller can take actions without human intervention. The challenge is to involve distribution systems with a large number of substations, distributed equipment/sensors, and energy resources, especially in locations where establishing a new communication infrastructure is not feasible.

System grids often promote a low energy consumption and a high penetration of local renewable energy generation in order to support future smart cities with sustainability programs including smart buildings [1,2,3]. This requires a direct interaction of distribution system customers with energy management and conservation entities [3]. However, the increased penetration of conventional and distributed energy sources, with the dynamic involvement of energy consumers, leads to more complicated distribution systems. As a result, profound changes in grid topology and operational schemes are eventually expected to be imposed [4]. Therefore, upgrading the distribution system communication network infrastructure to allow large data analysis and real-time operation and control strategies is essential to overcoming the foregoing challenges in establishing sustainable smart cities [1,3,5].

The success of achieving a high level of distribution system automation, such as predictive diagnosis for self-healing applications (i.e., automatic fault detection, localization, and power restoration), heavily relies on the availability and efficiency of the underlying communication infrastructure [6,7,8]. Exchanging all the sensed data and commands between the primary and distribution substations should be done reliably and in real time. Reliability implies communication system availability and its ability to guarantee the delivery of data packets. The real-time requirement is addressed by meeting a certain end-to-end delay. For instance, IEEE 1646 standard [9] provides guidelines for the time constraints to communicate information between intelligent electronic devices (IEDs) for substation integrated protection, control, and data acquisition. The standard classifies messages according to their urgency. For example, the maximum message delivery time required for system protection ranges from $\frac{1}{4}$ cycle (for applications within a substation) to 12 ms (for applications external to a substation) [9].

In light of the above, this work investigates the effectiveness of implementing HWNs by the aid of multipath transmission control protocol (MPTCP) in the communication infrastructure of future smart grid distribution networks. MPTCP uses multiple paths to deliver data simultaneously and reliably over multiple TCP subflows. This work proposes that distribution substations (either secondary or primary) be equipped with HWN-ready monitoring and control units (MCUs) with various integrated sensors. These MCUs are capable to communicate over hybrid wireless communication networks via dual-interface transceivers, supporting two different wireless technologies, and are MPTCP capable. The role of the MCUs is to collect data locally from a variety of sensors/intelligent electronic devices such as phasor measurement units (PMUs), current transformer meters (CTs) and potential transformer meters (PTs). An MCU establishes a single TCP connection (sub-flow) through each interface, forming two simultaneous TCP connections to exchange data among the substations (where different data packets are sent via each sub-flow). The objective behind utilizing MPTCP and hybrid communication technologies is to improve the overall throughput, reduce latency, and increase reliability. Indeed, aggregating the bandwidth of hybrid communication networks (e.g., WiFi and cellular 3G/4G) increases throughput and reduces the overall time needed to deliver a certain amount of data. Furthermore, in case high throughput is not a requirement, reliability is ensured as one interface can act as a redundant link at the lower communication layers, whereas the TCP protocol acknowledgment mechanism guarantees packet delivery at the upper layers.

The contributions of this paper are two-fold. First, we propose an HWN-based communication architecture for smart grid distribution networks that can achieve reliable and fast communication between primary and medium/low-voltage secondary distribution substations. We are interested in a hybrid communication architecture that relies on sending time critical grid information distributed over two different wireless network technologies. One is a contention-free licensed network that is based on a per-use cost structure such as 3G/4G networks. The other is a contention-based network running in a license-free industrial-scientific-medical (ISM) band that can be owned by the utility operator. Second, the performance of the proposed architecture is comprehensively studied by extensive computer simulations in realistic scenarios. The study addresses the performance gain and the effect of essential parameters (such as MPTCP maximum congestion window size, packet loss ratio, data rate, and burst size) on the overall link throughput and data transfer latency. It is worth noting that we do not address the tuning of MPTCP’s internal mechanisms; rather, we evaluate the overall HWN performance under different operational parameters to validate its suitability for distribution substations’ inter-communication requirements.

The rest of the paper is organized as follows. Section 2 reviews existing research works that are related to reliable communication architectures for distribution systems in smart grids. Section 3 introduces our system model from the viewpoint of future power distribution systems. Section 4 presents the proposed communication architecture and provides a brief overview of the MPTCP protocol. The performance of the proposed architecture is investigated in Section 5. Finally, Section 6 concludes the paper.

## 2. Related Works

Currently, many researchers are investigating employing wireless communication in various smart grid applications due to its easy deployment, low cost, and high supported data rates [10,11]. In [8], the author presents the design of sensor/data acquisition nodes that can be equipped with wireless transceivers in the system implementation of a predictive diagnosis scheme for safety-critical applications. Moreover, the authors in [3] discuss the need for the existence of sensor/control nodes with wireless capabilities that can be integrated into a building management system without disruptive changes to building controls. Indeed, the implementation of smart grid functions (such as predictive diagnosis for safety-critical applications [8], self-healing [12] or real-time system state estimation) at the distribution level mandates the exchange of a large data volume within a short period of time. This comes as a direct consequence of the very large number of distribution substations and/or monitored entities. Therefore, the underlying communication network needs to be highly reliable and fast. Due to the broadcast nature of wireless communications, identifying smart grid security vulnerabilities, attacks, and the mitigation of them are under focus of a multitude of research works as in [13] and the references therein. In addition, the authors in [14] present a comprehensive discussion and some proposed solutions to implement wirelessly-connected secure sensor/control nodes for a home area network, which represents an essential element in building smart power grids.

Few research works address the proposal of supporting the distribution level of power grids with advanced communication capabilities. Yang et al. [15] propose a satellite-based communication network to allow the exchange of control information among the 11 kV distribution substations in a single hop fashion. This infrastructure is nominally expensive, although the bandwidth is limited to only 9.6 kbps, which leads to a large latency. The author in [16] proposes a multihop wireless network with cellular frequency reuse as the communication infrastructure for low voltage distribution networks but without considering the reliability of data packet delivery.

Devidas et al. [17] study the ability of employing hybrid communication technologies (such as WiFi, Zigbee, cellular, and power line carrier) to carry various data types with different quality-of-service (QoS) requirements in a microgrid. The authors proposed a multi-tier heterogeneous network to direct data packets to the most appropriate communication technology for their QoS requirements. However, each tier runs only one communication technology. Kong [18] also studies the possibility of improving the QoS of grid data packets delivery. For this, a two-tier communication network combining the IEEE 802.15.4g standard and wired communication is proposed. This architecture, however, does not harness the capabilities of HWNs as it depends on disseminating sensed data wirelessly in the first tier by using only single-interface IEEE 802.15.4g-based devices.

Per packet end-to-end reliability is realized at the transport layer by the TCP protocol. In addition to congestion and flow control mechanisms, TCP also provides end-to-end error-free packet delivery. These TCP schemes are vital for the efficient utilization of bandwidth and prevention of packet loss [19]. In fact, network congestion causes deviations in phasor measurement units (PMUs) and IEDs’ real-time traffic, whose timeliness is essential in detecting and mitigating faults. Even with path bandwidth reservation, traffic congestion can still occur at times of increasing traffic demand and in transient traffic stress situations [20,21].

A proxy-like TCP mechanism is introduced in [22] as a solution to the TCP scalability concerns given the large number of data sources in a smart grid.

To the best of our knowledge, no other research work in the literature proposes and studies the performance of HWNs that employ MPTCP (as a transport layer protocol) in order to provide timely and reliable data transfer for smart grid distribution level applications.

## 3. Smart Grid Distribution System

Smart grids aim at realizing efficiency and reliability during different system operation modes. They allow advanced distribution management systems with remote controllability, whereas conventional distribution systems utilize local control algorithms [23]. Admittedly, data sharing among different elements in distribution systems is vital for smart decision-making. This requires transferring large amounts of data for real-time or non-real-time analysis according to the system operation mode and targeted function. However, the available communication infrastructures for distribution systems are not up to this challenge [24]. For instance, the SCADA systems of most utility companies do not monitor medium/low-voltage distribution substations.

**Figure 1:**
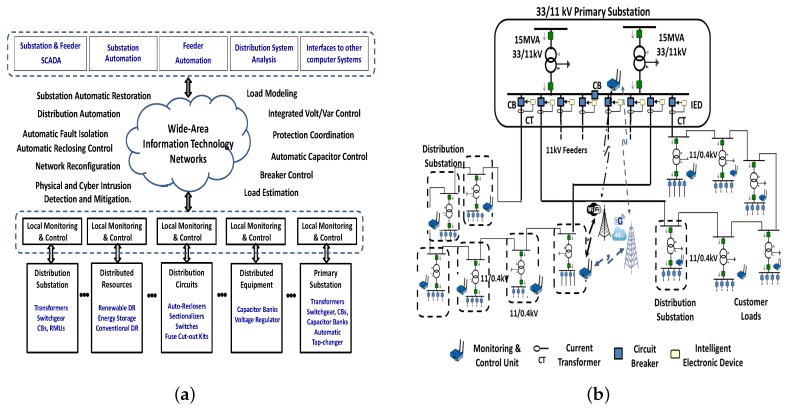
Future distribution system. (**a**) integrating local monitoring/control for future distribution functions; (**b**) a conventional distribution system upgraded towards a smart grid.

Figure 1 shows the main block diagrams of local/remote wide-area networks required for upgrading conventional distribution systems towards a smart grid. As illustrated in Figure 1a, the local monitoring and control units aim at continuous metering, control, and protection of main distribution system components. Several local monitoring and control units share information over a wide area network for remote controllability and implementation of different smart grid functions. The lower layer presents the typical physical components of a distribution system and the upper layer presents the centralized control system. The wide area and local communication networks are supposed to provide complete system integration that satisfies smart operation functions as shown in Figure 1a.

An example realization of the envisioned distribution system illustrated in Figure 1a is demonstrated in Figure 1b. The figure shows a part of an underground conventional 30/11 kV distribution system. The network configuration of conventional systems is typically radial in suburban and rural areas but looped in urban centers with high population density [1]. Compared with higher voltage level systems, there is a much lower level of network monitoring and automatic control that supports real-time operation. Intelligent electronic devices (IEDs) are located in the primary substations for monitoring, metering, control, and protection of outgoing feeders and primary substation transformers. Distribution substation automation can be enhanced by the deployment of smart meters/sensors that support large data set collection and metering functions.

In this paper, the conventional system is upgraded using monitoring and control units (MCUs) with integrated sensors for enhancing the operation monitoring and control. These MCUs are capable of communicating with one another over a hybrid communication network, which operates using two different wireless technologies. The upgraded distribution system model shown in Figure 1b is employed as a case study for investigating the advantages and challenges of HWNs in handling large data sets for real-time analysis.

In future smart grids, different levels of message classes (in terms of criticality and priority) should be analyzed and reported, in real-time or on-demand in order to realize different system functions. The required message delivery performance varies between a very high-speed (to develop a protection command) or a low-priority on-demand (for monitoring a quality of service). For example, a protection “Trip” command shall be delivered to other local IEDs within a total time of 4 ms or 5 ms for 50 Hz substations. However, fault recorders and power quality monitors, which are reported on demand, can deliver 64 to 256 samples/power system cycle with low priority within several seconds [9].

In self-healing applications, the proposed MCUs, via appropriate sensors, should process current/voltage signals, generate time tag synchrophasor measurement, and report in real time over the hybrid communication network via the MPTCP protocol to a central location at the primary substation. The synchrophasor (time tags) reporting rates are at sub-multiples of the nominal 50 Hz system frequency in the range of 10, 25 or 50 frames per second [25]. Typical systems have overall delay of synchrophasor reporting in the 20 ms to 50 ms range [26].

## 4. The Proposed HWN Architecture

In this section, we address the proposed communication architecture and shed light on the involved networks and the MPTCP protocol.

### 4.1. Inter-Networked Distribution Substations

Consider *N* distribution substations connected by feeders to a primary substation as depicted in Figure 1b. The large number of these substations mandates the usage of affordable but reliable communication technology. Our study focuses on wireless technologies since they are easy to install with cost-effective equipment, especially in old substations, where extending a wired infrastructure is hard. We assume that all the MCUs in the considered distribution substations can communicate to the MCU in the primary substation in a single-hop fashion as presented in Figure 1b (Only one connection between a distribution substation and the primary substation is shown for easy readability). Each MCU is equipped with a dual-interface wireless transceiver. One interface is a WiFi interface that offers a contention-based access using IEEE 802.11 protocol. The other interface connects to a 3G/4G base station directly. Since distribution substations serve the end user premises, we assume that all the *N* distribution substations and the primary substation are covered by a single WiFi network. This mimics a worst case scenario that can happen in densely-populated areas or in case the secondary substation role is similarly performed by a pole-mounted transformer. The assumed scenario also suits the case of the existence of a high capacity backbone network with a WiFi access at the edge. Indeed, this backbone can be represented either by a WiFi mesh network or WiFi-based data concentrators connected through a backbone network of a large capacity. In this case, the secondary substations compete for sending their data to the nearest mesh router or data concentrator, which forwards their data via the WiFi mesh or the concentrators’ backbone network to primary substations. The coverage of the WiFi network overlaps with a 3G/4G cellular network. Each 3G/4G link acts as a dedicated link with no packet contention. The cellular network interfaces for distribution substations are connected to the primary substation via the backbone of the cellular network, which is assumed to be of a high capacity with no limiting congestion.

The MPTCP protocol is used as the transport layer protocol. A brief overview of the MPTCP protocol is given in the next section.

### 4.2. Multipath Transmission Control Protocol (MPTCP)

MPTCP is one among several multi-homing protocols whose aim is to allow concurrent transfer of data over multiple interfaces. At the data link layer, channel bonding is implemented in IEEE 802.11 [27] to increase the bandwidth by combining two adjacent channels within a given frequency band. The technique suffers, however, from performance issues when the links have different rate and delay characteristics, i.e., the links belong to different communication technologies. Stream Control Transmission Protocol (SCTP), defined in RFC 4960 [28], was proposed as a multipath protocol able to run TCP’s congestion control on multiple sub-paths. The early version of SCTP, however, used only one path as a primary sub-connection, while the other interfaces were considered as backups. The newer version, Concurrent Multipath Transfer (CMT-SCTP) [29], has extended SCTP to utilize all the paths concurrently. Similar to CMT-SCTP, MPTCP is also designed to allow simultaneous use of multiple physical paths (heterogeneous or homogeneous) in the network, but it adds a management sub-layer at the transport layer. It has been shown experimentally in [30] that the MPTCP outperforms CMT-SCTP in most scenarios. Thus, it is selected as the transport layer protocol in our proposed network architecture.

MPTCP acts as a common transport layer that distributes the traffic to different networks in an HWN. Each network forms a sublayer that carries a single TCP connection (sub-flow). Therefore, the MPTCP layer controls how much data is pushed onto each subflow. Besides ensuring reliable data delivery by the usage of acknowledgments and retransmissions, the MPTCP provides flow control and congestion control functions for subflows similar to the TCP protocol. Flow control mandates that the amount of data to be transmitted should be less than the available space in the receiver buffer. Congestion control guarantees that the transmitted data size cannot exceed a certain limit (congestion window size) to be determined by a congestion control algorithm.

As the MPTCP main goal is to assign more data to the better subflow (i.e., the less congested), the criteria for traffic distribution among the underlying subflows has been investigated [31,32]. Several MPTCP implementations available in the literature are designed to resolve the negative impact that highly-congested subflows can cause to other subflows. Linked-increases algorithm (LIA) [33] has been implemented in some operating systems and is utilized in this work. LIA uses a parameter α to control the aggressiveness of a multipath flow. This congestion control algorithm works as follows.

For each acknowledgment (ACK) received on a subflow *i*, increase the subflow congestion window $w_{i}$ by

$$min(\alpha \times \frac{Bytes_{ACKed}\times mss_{i}}{w_{total}},\frac{Bytes_{ACKed}\times mss_{i}}{w_{i}}),$$

where $w_{total}$ is the sum of the congestion window sizes of all subflows in the connection and $mss_{i}$ is the maximum segment size on subflow *i*. The value of α can make a path aggressive, but, to ensure fairness, it is calculated such that it does not cause a subflow to be more aggressive than a competing TCP in a shared bottleneck. The parameter α is computed for each subflow considering the current properties of the flow as

$$\alpha =w_{i}\times \frac{max(\frac{w_{i}\times mss^{2}}{rtt_{i}^{2}})}{{\left(sum_{i}\frac{w_{i}\times mss_{i}}{rtt_{i}}\right)}^{2}},$$

where $rtt_{i}$ is the round trip time on subflow *i*.

MPTCP connections take place between the substations through dual-interface MCUs. The management sublayer of MPTCP initializes an end-to-end connection with MP-CAPABLE handshake. The two ends initiate the first subflow through one of the interfaces. The other subflow is established next using the MP-JOIN option signal over the other interface. After initialization, the MPTCP management layer distributes the data segments over the two links by means of a scheduling mechanism, whose objective is to provide load balancing, improve throughput, and achieve fairness.

## 5. Performance Study

In this section, the details of our simulation-based performance study are presented.

### 5.1. Simulation Setup

The simulated communication network considers one primary and seven secondary substations as depicted in Figure 1b. The simulation experiments are performed using the network simulator 2 (ns2) version 2.35, which implements the MPTCP protocol according to the IETF RFC 6824. Each substation has an MCU that is facilitated with two network interfaces, one of which is a WiFi connection and the other is a 3G/4G connection. It is assumed that all the distribution substations fall within the communication range of one another. The WiFi interface is configured according to Table 1, where all the configuration parameters are defined in [34]. The 3G/4G interfaces are simulated as dedicated wireless links between all the substations. The settings follow the peak data rates of different high speed packet access 3G/4G releases as listed in Table 2 [35].

A variety of scenarios and configuration parameters are examined. As a worst-case scenario, we investigate seven secondary substations simultaneously transmitting data packets to a primary substation for processing and analysis. Data transmission is managed by the MPTCP in order to distribute data packets over the two networks of the HWN. Data packets are 100 bytes in size. MPTCP adds a header of 60 bytes. For every received packet, the primary substation returns 40-byte ACK packets to every source. We evaluate the impact of various network configuration parameters, namely, the 3G/4G uplink speed, the maximum congestion window size, the amount of data to be collected from each substation, and the number of substations involved in the data transmission as listed in Table 3. The simulation results are mainly discussed in terms of the achievable throughput and the time duration to successfully collect a volume of data simultaneously from a number of substations.

### 5.2. Performance Gain of the Proposed Architecture

As mentioned earlier, transferring data over two separate communication networks strengthens the robustness of the system. By using MPTCP, if a link is down or congested, data is pushed on the link of the best condition. In the following experiment, seven substations transmit data continuously to the primary substation. The experiment addresses a scenario where the secondary substations are covered by more than one 3G/4G cell but sharing the same WiFi network. Thus, the MPTCP protocol is able to consume more capacity from 3G/4G cells as the ACK packets can be sent over both links in contrary to the case where only a WiFi or a 3G/4G link is available. The maximum congestion window $W_{M}$ of both WiFi and 3G/4G TCP subflows is 55 segments.

**Figure 2:**
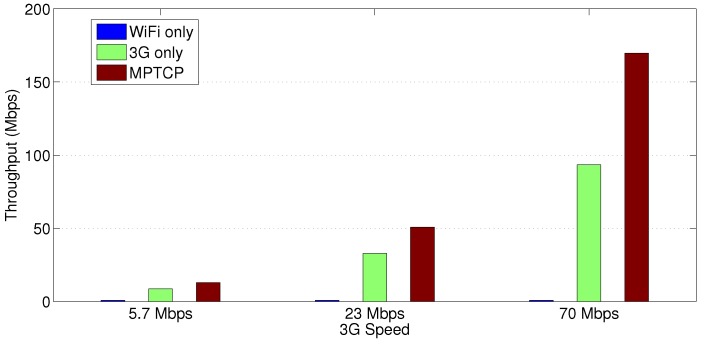
Throughput performance of single-path WiFi and 3G/4G versus MPTCP.

Figure 2 compares the data transfer throughput performance of the single-path WiFi and 3G/4G link cases versus our proposed HWN-based architecture using the MPTCP protocol. The figure clearly shows that the HWN configuration improves the throughput to a point better than any of the underlying technologies can achieve on its own separately. As Figure 2 reveals, relying only on the WiFi network leads to a significantly low throughput because all the eight substations, including the primary substation, share the wireless medium. As a matter of fact, the primary substation communicates back an ACK message for each segment submitted by the other substations. This greatly reduces the available bandwidth and increases the chance for collisions. In case of using a 3G/4G link alone, too many ACK packets accumulate at the transmission buffer of the primary substation’s transceiver (especially with high speeds). This leads to increased packet drops, although one ACK packet may acknowledge several received segments. On the contrary, a substantial improvement is noticed in Figure 2 with the HWN combining both networks using MPTCP. This is due to distributing the traffic between the interfaces according to the network congestion condition and link quality.

### 5.3. Impact of Available Contention-Free Link Capacity

**Figure 3:**
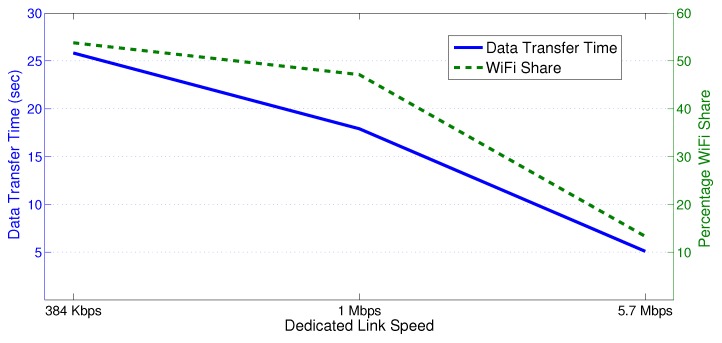
Data transfer time and the percentage of WiFi share.

Our proposed HWN architecture relies on MPTCP, which pushes more data on to the better link, i.e., the one with higher speed and lower packet loss. Transmission over WiFi leads to less throughput due to the medium access control (MAC) control messaging mechanism and the higher chance for packet collisions as many data sources compete for the media. On the other hand, the 3G/4G link can guarantee very low data transfer time since it is a contention-free link, but this comes at a higher cost for utility companies than WiFi networks. Here, we investigate how the available capacity of the 3G/4G link affects the data share that MPTCP allocates to each interface. Figure 3 shows the data transfer time and the percentage of WiFi share that result from delivering 500 KB data from seven substations to the primary substation. The connections are configured such that the congestion window size $W_{M}$ equal to 55 segments for each subflow. In essence, wireless channel impairments cause packet loss. Here, we assume that both links have a packet loss ratio (PLR) of 0.005. The 3G/4G link speed is varied from 384 kbps to 5.7 Mbps. As Figure 3 depicts, increasing the 3G link speed leads to a significant decrease in the amount of data transferred over WiFi (percentage WiFi share). Apparently, MPTCP gives preference to the contention-free link over WiFi even if the link speed is comparable to the available WiFi per-substation channel capacity. This happens mainly due to packet collisions.

### 5.4. Effect of MPTCP Maximum Congestion Window Size

**Figure 4:**
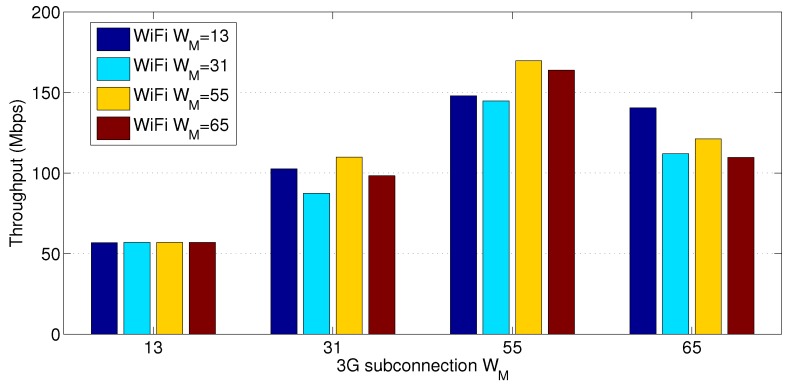
Throughput comparison at 3G speed 70 Mbps.

The maximum congestion window $W_{M}$ is an essential parameter for tuning the MPTCP protocol performance. We found that the selection of $W_{M}$ leads to a significant change in the achievable throughput. Figure 4 shows the MPTCP throughput performance for different combinations of $W_{M}$ values for the WiFi link and a 70 Mbps 4G link. In fact, as Figure 4 reveals, there is some optimal value of $W_{M}$ that gives the best throughput. For example, at a 3G speed of 70 Mbps, setting the $W_{M}$ of both interfaces to 55 segments gives the highest throughput. Determining the right value of $W_{M}$ for an interface is subject to a further study considering a number of related parameters, such as $W_{M}$ of the other interface, speed, buffer capacity, and the number of competing devices. The reason for the variation of throughput is that a large $W_{M}$ allows the congestion window to grow to a large value. This implicitly means sending more segments on each interface, and hence increasing the throughput. However, in our network setup, the primary substation has to send ACK messages to every substation. Therefore, as the congestion window size of a sending interface becomes large, the buffer size of the corresponding receiving interface at the primary substation overflows quickly. As a result, a large number of ACK packets are dropped causing throughput degradation. On the other hand, small values of $W_{M}$ do not lead to dropping large number of ACK packets at the primary substation but lead to a low throughput.

### 5.5. Network Performance with Different Data Sizes

**Figure 5:**
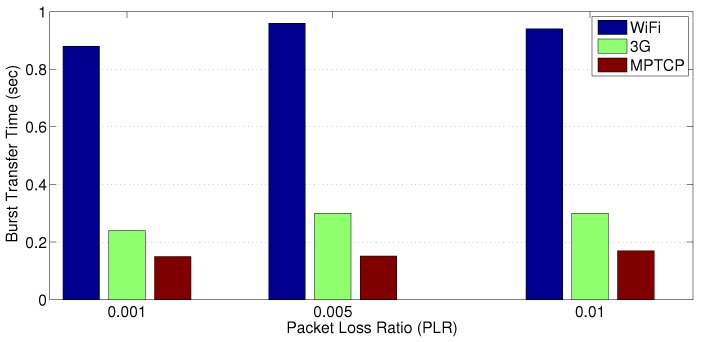
A comparison of the transfer time of a 5 KB burst for different PLRs.

In this section, we investigate a scenario where a number of distribution substations suddenly have to report some amount of information (a data burst) to a primary substation. This scenario reflects the need for these substations to share urgent sensed data (e.g., data describing a phenomena for self-healing purposes or real-time system state estimation) with the primary substation. Our study for this scenario takes into account different PLR values for both links (WiFi and 3G/4G). Figure 5 shows the average data transfer time of a 5 KB data burst using the WiFi network alone, a 1 Mbps 3G link only, and by using HWN over both networks. Apparently, the HWN via MPTCP offers the best data transfer latency performance for utility companies.

**Figure 6:**
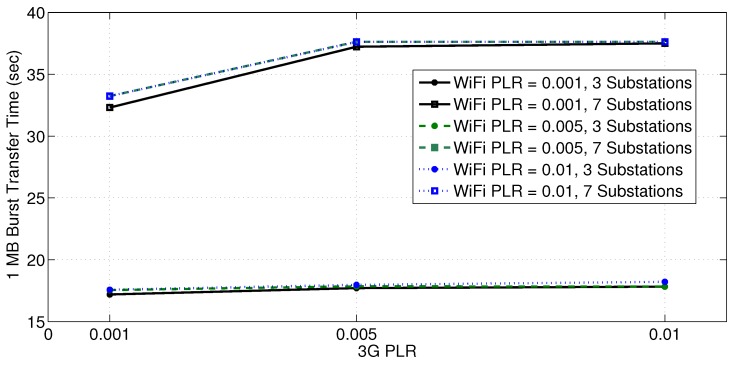
Data burst transfer time for different number of substations and PLRs.

In another experiment, the 3G/4G link capacity is kept at 1 Mbps, while only three substations simultaneously report a 1 MB of data to the primary substation at different combinations of PLRs (for both interfaces) using MPTCP. Figure 6 clearly reveals a significant reduction in the data transfer time as the number of substations exchanging the data bursts decreases. This is mainly due to the availability of a higher wireless channel capacity per substation and the lower chance of packet collisions. The figure also shows that different PLRs have a slight effect on the burst transfer time for the 3-substations case. For the 7-substations case, the effect of PLR is a bit more pronounced due to the larger data volume exchanged. Thus, the distribution system operator should keep the number of substations involved in simultaneous data reporting as low as possible, if low data transfer latency is required.

**Figure 7:**
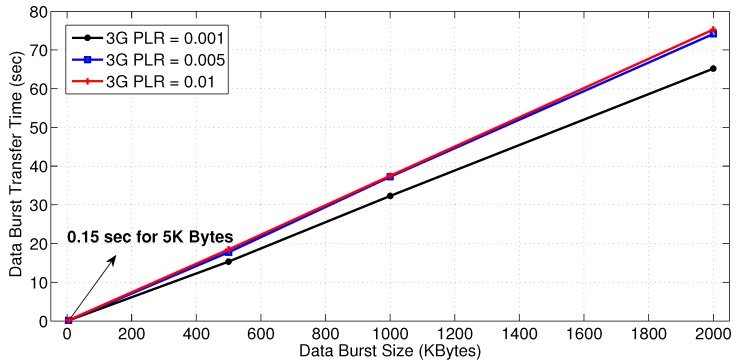
Data burst transfer time with different burst sizes and 3G PLRs.

The data transfer time of the proposed architecture is evaluated and showed in Figure 7 for different data burst sizes for a WiFi PLR of 0.001 and different PLRs for a 1 Mbps 3G link. All the data bursts are sent simultaneously from the seven substations to the primary substation. As depicted in the figure, the data transfer time is almost linearly increasing with the burst size when using a HWN. A 5 KB burst takes 0.15 s, whereas a 500 KB burst takes 15 s. This makes the data transfer time for other burst sizes easy to be interpolated or extrapolated for the same network parameters. Thus, Figure 7 serves the general purpose of providing the relation between the size of transferred data and latency for inter-substation communication. This fits the applications that require the exchange of data messages in order to transfer information between substations (e.g., tele-protection or phasor measurements) [36]. For instance, IEEE C37.118 [37] defines two performance classes. The first is the P Class. It addresses the applications that require fast response such as protection applications where data transfer should be in the form of small packets in order to satisfy real-time requirements. The other class is the M Class. It targets the applications that require greater precision but not restricted to minimal reporting delay such as asset management, situation awareness, and power quality monitoring [37]. Therefore, this class is based on burst data reporting, which is essential for accurate analysis [37]. Accordingly, the figure provides the general case of transferring data in different packet sizes not necessarily in bursts.

### 5.6. HWNs for Future Distribution System Applications

**Figure 8:**
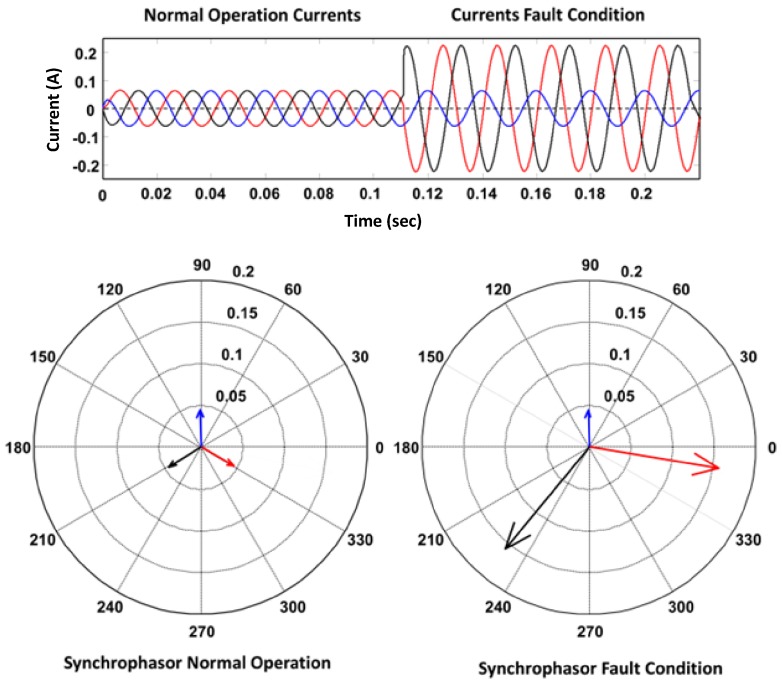
Current waveforms and synchrophasor measurements during normal operation and a fault condition.

Figure 7 can provide some guidelines about the ability of the proposed architecture to satisfy real-time constrains in some future distribution system applications. As an example, we show here that the HWN-based architecture is capable of supporting an automated fault-detection application for grid self-healing purposes. The three phase current waveforms and the synchrophasors generated during normal and fault conditions are illustrated in Figure 8. A double line-to-ground fault (ABG) occurs at time instant 0.11 s as detected on the current waveform. Based on a reporting rate of 50 frames per second, the synchrophasor measurements during normal operation are reported every complete cycle (0.02 s) at time instants 0.06, 0.08, and 0.01 (in seconds) and at time instants 0.12 up to 0.22 s during fault condition. Due to a stable 50 Hz frequency, the synchrophasors show a steady change in the magnitude and no change in the angle. These synchrophasors can be used to automatically detect and report the fault condition.

In order to investigate the performance of our proposed architecture in this application, a reporting rate of 10 frames per second is selected. The proposed MCUs of the distribution substations are set to continuously report every five complete cycles (100 ms). These units should report eight synchrophasor measurements (three-phase line and neutral currents, three-phase voltages, and rate of change of frequency) each of 4 bytes in size with a speed of 10 samples per second. This results in a data packet size of 320 Bytes for each substation. These data packets can be transferred simultaneously from the seven substations within an end-to-end delay of 9.6 ms using the proposed HWN configuration. Recently, IEC 61850-90-1 has defined the communication requirements (in terms of message types and transfer times) between substations [36,38]. The obtained end-to-end delay (9.6 ms) meets the most stringent delay requirement for IEC 61850-90-1, which is assigned to message performance class (MPC) Type 1 and Type 4 (maximum 10 ms transfer time) [38] according to Table 4. Indeed, the packet size for these message types tends to be smaller, and hence can be transferred in a shorter time using the proposed architecture. Moreover, this estimated end-to-end delay also sufficiently meets the delay requirements of different PMU wide area monitoring and control applications [26,39].

Generally, data transfer times of similar order of magnitude or smaller can be achieved for larger packet sizes by increasing the 3G data rate as revealed in Figure 3. However, this comes at a higher operation cost. Therefore, the data size and the number of involved substations should be controlled by the system operator in order to report this data within the required time constraint.

### 5.7. Discussion

Our performance study demonstrates that using HWNs is effective in reducing the data transfer latency. It also shows that HWNs can play an essential role in increasing the reliability of network connections against packet loss. However, supporting the substations with a cellular technology (3G/4G) may increase the overall cost of the proposed infrastructure, if it is not carefully managed. Apparently, it is better to utilize the WiFi infrastructure to its full capability and keep the cellular bandwidth subscribed or the data usage plan as low as possible. In this section, we discuss a limitation of the capability of HWNs, which is the ability of the MPTCP congestion control algorithm to maximize the utilization of the WiFi network.

**Figure 9:**
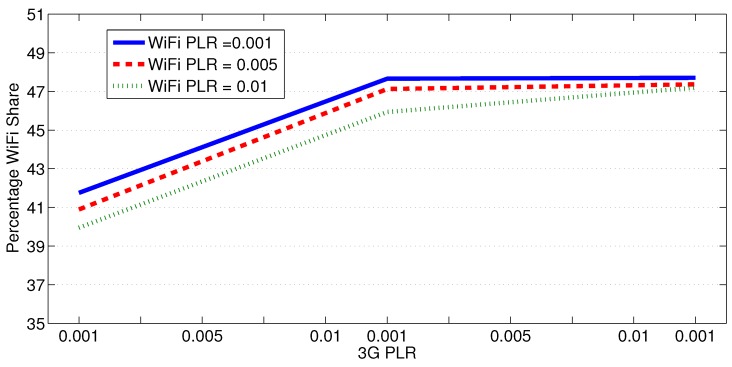
Traffic distribution for subflows with different PLRs.

Generally, MPTCP tends to push more traffic on the subflows that suffer less packet loss, irrespective of if this loss is due to congestion, channel impairments, or other reasons. Figure 9 shows how the WiFi traffic share changes as one of the links becomes lossy. In this experiment, a 1 Mbps 3G link is used with the WiFi network to deliver a 500 KB of data from each one of the seven secondary substations to the primary substation simultaneously. It is clear that, at any PLR of the 3G link, as the loss rate of the WiFi link increases, more data is pushed over the 3G link. However, as the PLR of the 3G link increases, the WiFi share does not exceed 48%.

**Figure 10:**
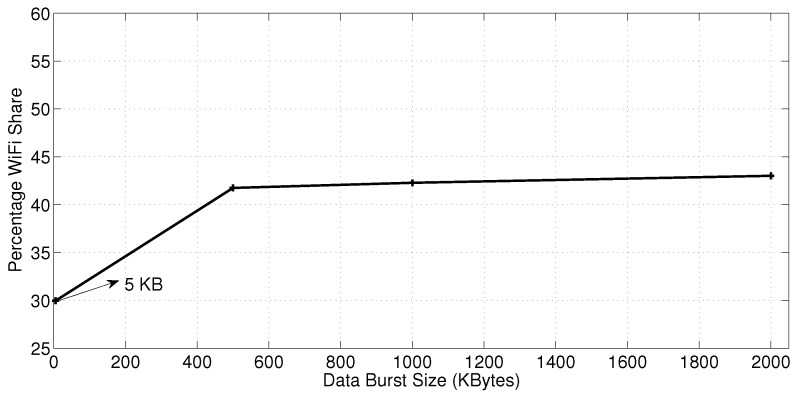
WiFi share with different burst sizes.

Figure 10 shows the effect of the data burst size on the WiFi share. The values are measured for a 1 Mbps 3G link transferring, in conjunction with the WiFi network, a 1 MB of data with 0.001 PLR for both the 3G and the WiFi link. Again, Figure 10 reveals that the MPTCP pushes at maximum only around 44% of the data traffic over the WiFi link. For small burst sizes, such as 5 KB, the share can be as low as 30%. The reason for this trait is the existance of packet collisions that WiFi networks suffer from. Admittely, packet collisions add to the channel packet loss rate. This makes a 1 Mbps 3G link more preferable to the MPTCP congestion control algorithm, although the eight substations (including the primary) are sharing a 54 Mbps WiFi network bandwidth.

**Figure 11:**
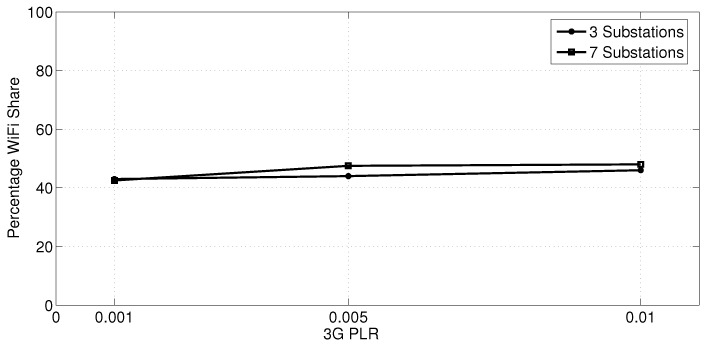
Percentage WiFi share for three and seven substations with different 3G PLRs.

In another experiment (with the same parameters as the previous one), we measured the WiFi traffic share when only three substations are involved in transferring a 1 MB data burst simultaneously. As depicted in Figure 11, there is almost no difference in the WiFi share between the 3-Substations case and the 7-Substations case. This indicates that the congestion control algorithm is not able to increase the WiFi share for the same 3G/4G link rate, although more bandwidth is available per substation in the 3-Substations case. In fact, the congestion control algorithm is capable of increasing the WiFi share only if the data rate of the 3G/4G link is reduced to 384 kbps as shown in Figure 3, but the WiFi share barely exceeds 50%.

## 6. Conclusions

In this paper, the performance of a proposed HWN-based architecture in a typical data exchange scenario of future smart grid distribution system is studied. The introduced scenario examines the ability of the proposed architecture to exchange data bursts in real time from a number of distribution substations to a primary substation. We investigated the data transfer performance of proposed network configuration (using the MPTCP protocol) over two wireless links of different technologies, namely, WiFi and cellular (3G/4G), by using extensive computer simulations. Our findings demonstrate that HWNs can achieve reliability (for different packet loss ratios) with low data transfer latency as mandated by the requirements of distribution system standards. On the other hand, our findings also reveal that the MPTCP, as a candidate transport layer protocol, tends to push more data on 3G/4G links than WiFi. This happens in case the 3G/4G link is of comparable or higher capacity than the available WiFi capacity per substation. Although this increases the HWN-based solution cost compared with using only WiFi networks, it offers better reliability and lower cost than using cellular network technologies alone given the achieved low data transfer latency.

## Figures and Tables

**Table 1:** IEEE 802.11 System parameters.

System Parameter	Value
Basic Rate	6 Mbps
Data Rate	54 Mbps
PHY	20 μs
PLCP	24 bits
MAC Overhead	246 bits
$T_{ACK}$	22.3 μs+ PHY
$T_{RTS}$	30.3 μs + PHY
$T_{CTS}$	22.3 μs + PHY
Slot Time	9 μs
SIFS	16 μs
DIFS	34 μs
$CW_{min}$	32
$CW_{max}$	1024

**Table 2:** 3G/4G maximum speeds.

3GPP Release	Downlink Speed (Mbps)	Uplink Speed (Mbps)
Rel 6 (3G)	14.4	5.7
Rel 9 (4G)	84	23
Rel 11 (4G)	336–672	70

**Table 3:** Configuration parameters.

3G/4G speeds	384 kbps, 1 Mbps, 5.7 Mbps, 23 Mbps, & 70 Mbps
Max congestion window size	13, 31, 55, & 65
Data burst size	5 KB, 500 KB, 1 MB, & 2 MB
Number of substation	3 & 7

**Table 4:** IEC 61850-90-1 transfer time requirements [36,38].

Message Type	Application	Transfer Time (ms)
1A	Fast messages (e.g., Trip)	3–10
1B	Normal/Other fast Messages	20–100
4	Raw Data	3–10
2	Medium speed	100
3	Low speed	500
6	File transfer	1000
